# Protocol for inoculating bottled mineral water with bacteria to receive artificial contaminated water samples

**DOI:** 10.1016/j.xpro.2024.103026

**Published:** 2024-04-30

**Authors:** Hans-Peter Ziegler, Sabine Platzer, Doris Haas, Herbert Galler, Michael Schalli

**Affiliations:** 1 Department for Water-Hygiene and Micro-Ecology, D&R Institute of Hygiene, Microbiology and Environmental Medicine, Medical University of Graz, 8010 Graz, Austria; 2 Applied Hygiene and Aerobiology, D&R Institute of Hygiene, Microbiology and Environmental Medicine, Medical University of Graz, 8010 Graz, Austria

**Keywords:** Cell culture, Chemistry, Microbiology

## Abstract

Here, we present a protocol for inoculating drinking water samples with a variety of pathogens or facultative pathogen bacteria. We describe steps for preparing bacterial solutions, inoculating mineral water bottles and other drinking water samples, filtration and incubation of the agar plates, and counting colony-forming unit per mL. We also detail procedures for determining selected chemical properties, such as anions and cations, which can also affect the bacterial growth.

For complete details on the use and execution of this protocol, please refer to Schalli et al.^1^

## Highlights

• Detailed steps to artificially contaminate bottled mineral water
• Steps to analyze the chemical properties of water
• Steps for filtration and incubation of agar plates and validation of strains

## Abstract (editor highlights)

Publisher’s note: Undertaking any experimental protocol requires adherence to local institutional guidelines for laboratory safety and ethics.

## Graphical abstract

**Figure:**
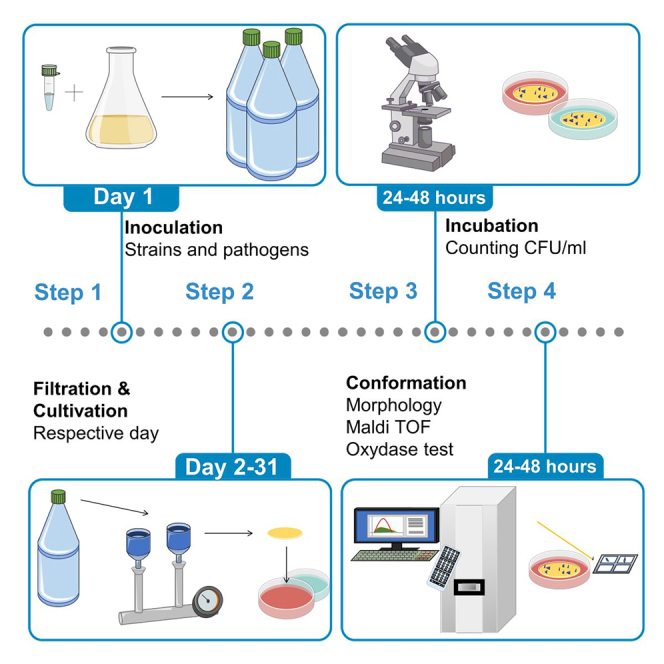

## Before you begin

The protocol below describes a method of inoculating drinking water samples with a variety of pathogens or facultative pathogen bacteria. We describe steps for preparing bacterial solutions, inoculating mineral water bottles and other drinking water samples, filtration and incubation of the agar plates, and counting CFU/mL. We also detail procedures for determining selected chemical properties, such as anions and cations, which can also affect the bacterial growth.

### Institutional permissions

All experimental procedures require compliance to the safety guidelines of the institution or laboratory.

### Preparation one: Contaminant solution and incubators

**Timing: 30–60 min**

1. First, prepare the contamination solution to inoculate the water samples.
  a. Autoclave a sterile glass bottle containing 1000 mL of distilled water at 121°C for 20 min for each strain.
  b. Dilute one ceramic bead in a bottle of distilled water (1000 mL):
  c. Gently mix the solution by inverting the bottle.
  d. After 10 min the contaminate solution is ready.
  e. To determine the CFU/mL in the contaminated starting solution, prepare a serial dilution and plate it on tryptic soy agar. Afterwards count your plates and note the CFU/mL you started with.
2. For the incubation step of the artificial contaminated water at the period of 31 days set the incubator to 25°C
3. The incubators should be set to 37°C to provide an optimal growth rate for counting the CFU/mL of the strains.

***Note:*** This protocol requires bacterial strains to inoculate the water samples. The strains we used were obtained from commercial sources (*E. coli* DSM 1103 and *P. aeruginosa* DSM 50071, Leibnitz-Institute, DSMZ-German Collection of Microorganisms und Cell Cultures GmbH, Braunschweig, Germany), which were shipped in glass vials and contain freeze-dried material on ceramic beads. More important, we started the protocol using the beads covered in a cryopreservative solution with around 10^6^ CFU/mL. The CFU/mL of the beads covered in the cryopreservative solution can be found in the datasheet of the respective strains that are listed in the table REAGENT or RESOURCE.

### Preparation two: Equipment for the filtration

**Timing: 10–15 min**

4. For this protocol, take a cylindrical funnel with a volume of 100 mL and filtrate an amount by 10 mL–100 mL of the artificial contaminated water samples.^2^^,^^3^
5. To verify Enterobacteriaceae filtrate the 100 mL of the artificial contaminated water and put the filter on Endo agar plates.

***Note:*** For the filtration of the water samples through a porous membrane, filtration equipment is necessary.

**CRITICAL:** The handling of the filtration equipment can adversely affect the efficiency of this technique. Furthermore, the right handling avoids cross contaminations to receive reliable results.

***Note:*** The membrane disc must have the correct pore size of 0.45 μm to ensure the right strains are filtered.^3^

***Note:*** If you store the plates at 4°C in the fridge, be sure to preheat to 25°C before it's usage.

### Preparation three: Eluent for ion chromatography and reagents for photometry

**Timing: 45 min**

6. To prepare the anion eluent add 42.4 g Na_2_CO_3_ and 4.2 g NaHCO_3_ to 500 mL deionized water to reach an eluent concentrate of 800 mM Na_2_CO_3_ and 100 mM NaHCO_3_.
  a. Freshly prepare the eluent using 20 mL of the solution prepared in 1. a. and dilute in 1980 mL deionized water.

***Note:*** We use the stock solution to prepare the eluent for the ion chromatography ICS 1100.

7. Dissolve 260 g C_7_H_5_NaO_3_ and 260 g Na_3_C_6_H_5_O_7_ in 1600 mL deionized water.
  a. Once the chemicals are dissolved, add 1.94 g sodium nitroprusside and mix the reagent again to obtain reagent 1.
8. The reagent 2 contains 64 g sodium hydroxide in 1000 mL deionized water.
  a. Add 4 g sodium dichloride isocyanurate and mix the solution until all chemicals dissolve.

***Note:*** The photometric determination of the ammonium values in the samples requires two reagents.^4^

9. Add 100 mL Orthophosphoric acid to 500 mL deionized water, to prepare the reagent to measure the nitrite.^5^
  a. Add 40 g sulfanilamide to the orthophosphoric acid solution and mix it well.
  b. Continue to get the reagent by adding 2 g naphthylethylenediamine dihydrochloride.

***Note:*** We use the Griess test to measure the nitrite ions in the water samples.

## Key resources table

**Table:** 

REAGENT or RESOURCE	SOURCE	IDENTIFIER
**Chemicals, peptides, and recombinant proteins**		

Sodium carbonate	Roth	Cat#A135.2
Sodium hydrogen carbonate	Merck GmbH	Cat#1.06329
Argon 99.99%	Fa. Air Liquide	Cat#231-147-0
Sulfuric acid 96%	Merck GmbH	Cat#1.00714
Sodium peroxidisulfate	Merck GmbH	Cat#1.06609
Orthophosphoric acid	Merck GmbH	Cat#1.00573
Nitric acid conc.	Merck GmbH	Cat#1.00441
Sodium citrate	Merck GmbH	Cat#1.6448
Sodium nitroprusside	Merck GmbH	Cat#1.06541
Sodium salicylate	Merck GmbH	Cat#1.06601
Sodium hydroxide	Merck GmbH	Cat#1.06498
Sodium dichloride isocyanurate	Merck GmbH	Cat#1.10888
Sulfanilamide	Merck GmbH	Cat#1.11799
Naphthylethylenediamine dihydrochloride	AppliCem	Cat#A2782

**Bacterial and virus strains**		

*E. coli*	DSM	DSM 1103
*P. aeruginosa*	DSM	DSM 50071

**Biological samples**		

Bottled mineral water		

**Deposited data**		

Raw data	Supporting information, https://doi.org/10.1016/j.heliyon.2023.e21634	

**Other**		

Tryptic soy agar (TSA)	VWR	Cat#101114ZA
Endo agar	Merck GmbH	Cat#104044
Cellulose ester filter, 47 mm diameter, 0.45 μm pore size	Merck GmbH	Cat#EZHAWG474
DrySlide oxidase 231746	BD	Cat#231746
MALDI-TOF VITEK MS	bioMerieux	
Memo-Titrator	Metrohm	Titrando 888
LL Aquatrode Plus	Metrohm	Cat#6.0253.100
Conductometer	Metrohm	712
TOC Multi N/C	Analytik Jena GmbH	N6-581/M
ICP-OES	Thermo Fisher Scientific, Inc.	ICAP7000 Plus
Dionex ICS	Thermo Fisher Scientific, Inc.	ICS-1100
Dionex IonPac AS14A IC columns	Thermo Fisher Scientific, Inc.	Cat#056904
Dionex IonPac AG 14 P/N columns	Thermo Fisher Scientific, Inc.	Cat#56904
Spectrophotometer	Hitachi	U-2900
150 mL beaker	FA. Nalgene	
Stainless steel funnel for vacuum filtration	Merck GmbH	XF2014752
Vacuum filtration manifold	Merck GmbH	XX2504700
Heraeus incubator	Thermo Fisher Scientific, Inc.	B 6200
RuMed incubator	Thermo Fisher Scientific, Inc.	D/30-36/02-2001
Autoclave Systec	Bartelt GesmbH	VX-120

## Materials and equipment

**Table:** Stock solution for ion chromatography

Reagent	Final concentration	Amount
ddH_2_O	N/A	500 mL
Na_2_CO_3_	400 mM	42,4 g
NaHCO_3_	50 mM	4,2 g
**Total**	**N/A**	**500 mL**

***Note:*** The eluent concentrate can be stored for 2 months at 25°C. Further chemicals that we used in this protocol, e.g., acidifying samples, are listed in the key resources table.

**Table:** UV/VIS solution (reagent 1)

Reagent	Final concentration	Amount
C_7_H_5_NaO_3_	385 mM	260 g
Na_3_C_6_H_5_O_7_	620 mM	260 g
Na_2_[Fe(CN)_5_NO] ∗ 2 H_2_O	4 mM	1.94 g
ddH_2_O	N/A	1600 mL
**Total**	**N/A**	**1600 mL**

***Note:*** If you keep reagent 1 in the fridge at 4°C, the solution can be stored for 2 weeks. Contrariwise, freshly prepare reagent 2 every day.

**Table:** Griess Solution

Reagent	Final concentration	Amount
ddH_2_O	N/A	500 mL
H_3_PO_4_	1 M	100 mL
C_6_H_8_N_2_O_2_S	387 mM	40 g
C_12_H_14_N_2_	18 mM	2 g
**Total**	**N/A**	**600 mL**

***Note:*** The reagent for Griess test is stable for 1 month, since it's stored in the fridge at 4°C. If you want to store the solution up to one year, keep the reagent at −25°C.

## Step-by-step method details

### Step one: Analyses on the chemical properties

**Timing: 1–1.5 h**

Follow this procedure to determine the chemical properties of mineral water (Table 1) without carbon dioxide and with additional CO_2_.

1. For analyzing the pH fill a 150 mL beaker with 100 mL of the respective water sample and run the program to measure the pH value at RT.^6^
2. To get the conductivity score use the same beaker filled with 100 mL of the water sample and do the measurements with the conductometer.^7^

***Note:*** We use the Metrohm Tritator connected to the sample changer 730 to move the samples. For this reason, we measured the pH and conductivity values of the samples consecutively and the raw data was processed using the Tiamo software.

3. Add 50 μL sulfuric acid (2M) with additional 80 g/L sodium peroxidsulfate to the reaction tube and put the sample into the TOC analyzer.^8^ Troubleshooting 1.

***Note:*** You have to ensure that the argon gas flow is set between 130 and 150 mL/min.

4. To assay the dissolved metal ions, take 100 mL of the water samples, filter it through a 0.45 μm membrane and add 1 mL of HNO_3_ conc. to acidify the sample for the determination of Fe, Mn, Ca, Mg, Na and K.^9^
  a. Put the samples to the ICAP 7000 sampler module and start the program.
  b. Calculate the hardness by the values of Calcium and Magnesium. DIN 38409:1986:01 DEV H6.

***Note:*** We use the samples changer ASX-280 for the ICP-OES to analyze the water samples in serial, applying the Qtegra software version 2.8.2944.202 to calculate the results and values for each ion.

5. For mineral water samples, please prepare a 1 to 10 dilution and set it in the schedule. Put the vial to the sample holder and run the program.

***Note:*** Our ICS-1100 is connected to AS-DV sample changer to analyze the samples subsequently. Once the program is finished, Chromeleon Version 7.2.10.ES software evaluates the anion values. To determine the anions (Nitrate, chloride and sulfate) using the ion chromatography run the correct program on the Dionex ICS-1100.^10^ Before you start with the samples rinse the system with the eluent solution to receive a linear baseline. Troubleshooting 2.

***Note:*** We measured the ammonium and nitrite ions using a spectrophotometer.^4^

6. Take a reaction tube and fill in 5 mL of the water sample to measure the ammonium ions.
  a. Add 0.4 mL of the previously prepared reagent 1 to the water sample and mix it well.
  b. Additionally, add 0.4 mL of freshly prepared reagent 2 and mix the reaction tube. The pH of the samples should now reach 12.6.
  c. Measure the extinction at a wavelength of 665 nm to get the ammonium values.
7. To measure the value of the nitrite ions add one drop of the Griess reagent to the samples (5 mL).^5^
  a. Wait for 20 min until the reaction is finished.
  b. Set the wavelength to 540 nm and measure the extinction.

***Note:*** The calculation of the values was done after the procedure mentioned in ÖNORM ISO 7150-1:1987 12 01 (DIN 38406-5:1983 (DEV-E5))

**Table 1:** The chemical properties of mineral water with and without CO_2_

**Chemical properties of bottled water without additional CO**_**2**_					

Calcium (Ca^2+^)	Magnesium (Mg^2+^)	Sodium (Na^+^)	Potassium (K^+^)	Iron (Fe)	Manganese (Mn)
54.2	14.1	21.5	1.7	<0.02	<0.05
Ammonium (${\text{NH}}_{4}^{+}$)	Nitrite (${\text{NO}}_{2}^{-}$)	Nitrate (${\text{NO}}_{3}^{-}$)	Chloride (Cl^-^)	Sulfate (${\text{SO}}_{2}^{-}$)	Hardness (°dH)
<0.02	<0.01	10.1	18.6	17.1	10.8

**Chemical properties of sparkling bottled water**					

Calcium (Ca^2+^)	Magnesium (Mg^2+^)	Sodium (Na^+^)	Potassium (K^+^)	Iron (Fe)	Manganese (Mn)
53.5	14.2	20.5	0.7	<0.02	<0.05
Ammonium (${\text{NH}}_{4}^{+}$)	Nitrite (${\text{NO}}_{2}^{-}$)	Nitrate (${\text{NO}}_{3}^{-}$)	Chloride (Cl^-^)	Sulfate (${\text{SO}}_{2}^{-}$)	Hardness (°dH)
<0.02	<0.01	10.6	21.8	18.2	10.7

The values in the units [mg/L] for the chemical components and the hardness is calculated in German hardness degree [°dH].

### Step two: Inoculation of the water samples

**Timing: 30–45 min**

To prepare the contaminated water samples, following steps are mandatory.

8. Set up 3 water bottles for each condition and pathogen, e.g., Figure 1.
  a. Open a fresh water bottle or your sample and inoculate with 1 mL of the contaminant solution, you have prepared previously.
  b. Immediately close the bottles again and then gently mix your water samples by orbital movements of the bottle. Troubleshooting 3.

**Figure 1:**
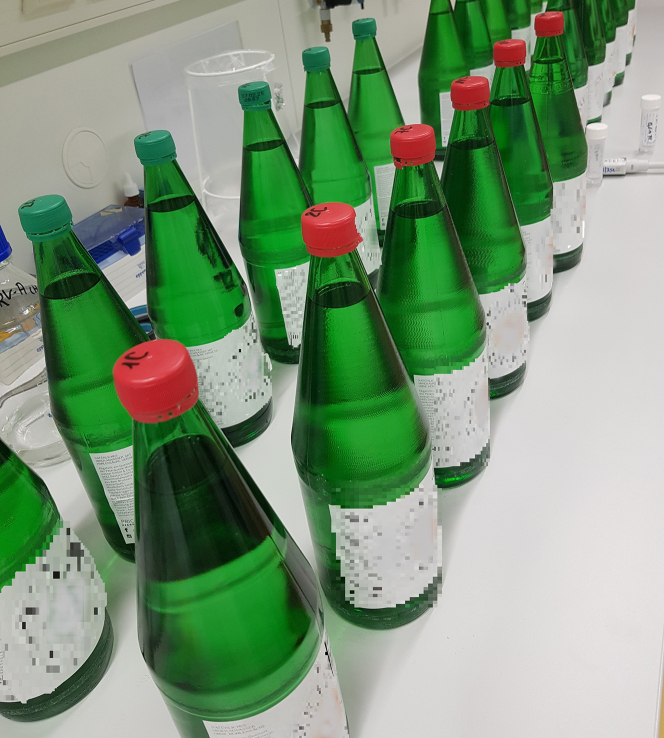
Inoculating mineral water bottles with and without carbon dioxide An example of commercial sparkling mineral water bottles (green capped) and ones without carbonization (red cap) that we used in this investigation.
9. Put the artificial contaminated water samples to incubation at RT for 1–31 days (dark place).

**CRITICAL:** Due to the pathogenicity of the used strains (key resources table), please be careful during the preparation procedure.

***Note:*** Once you have inoculated all your samples, prepare non-artificial contaminated samples to determine your background-flora.

***Optional:*** If you want to do any investigations on the background-flora in artificial contaminated water, prepare the samples for the same period.

### Step three: Filtration and cultivation over a period of 31 days

**Timing: 1 h**

**Figure 2:**
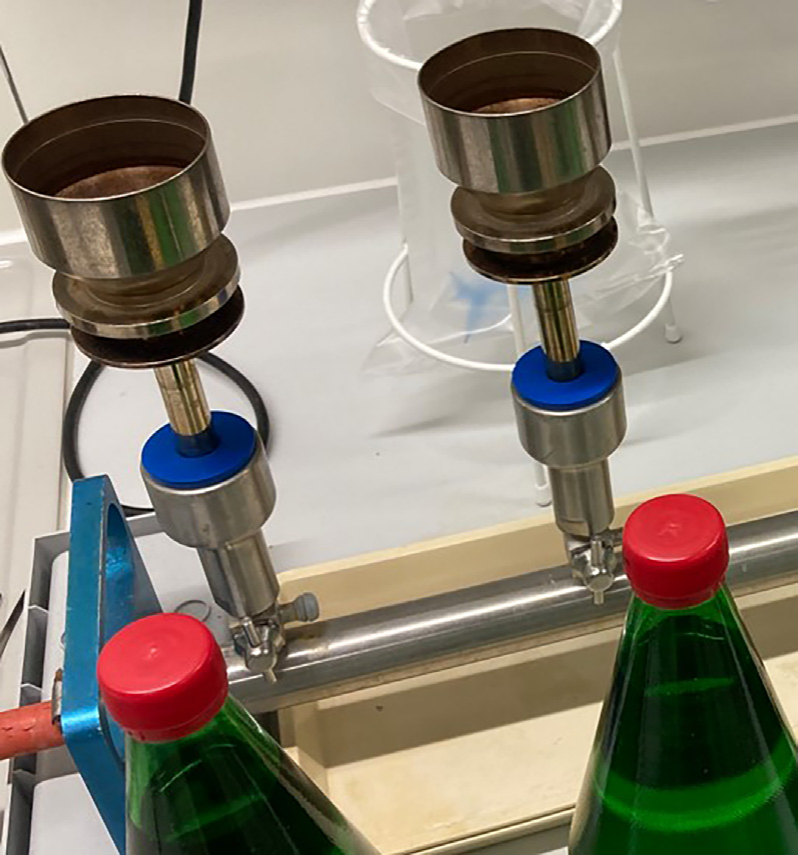
Make sure, that the sterilized filtration equipment is ready

After incubating the water bottles for 1 day (24 h), 2 days, 7 days, 2 weeks, 3 weeks and 31 days proceed with the filtration to get the CFU/mL over this period.^3^^,^^11^^,^^12^ After Filtration (Figure 2) and incubation of the agar plates count all the colonies on the plates to calculate the CFU/mL.

**Figure 4:**
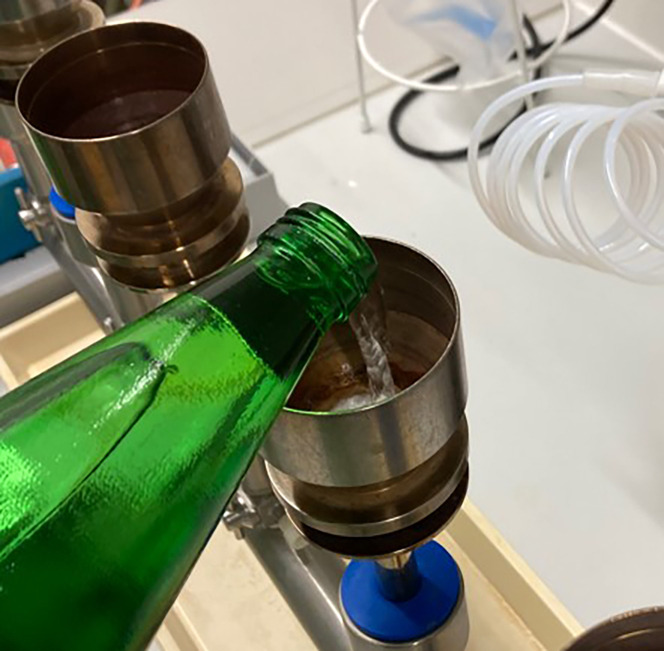
Fill the respective amount of sample into the prepared apparatus

10. First, make sure that the equipment, which you have prepared before, is ready to use and open the bottle at the respective day (e.g., Figure 3).
  a. Place the filter on the cylindrical funnel and check that the funnel top is not leaking out, otherwise you will lose the feed or get no vacuum to filtrate sufficient (Figure 3).
  b. Fill in 100 mL of the inoculated water samples and filter it through the cellulose ester filter using vacuum (Figure 4).^2^^,^^3^

**Figure 3:**
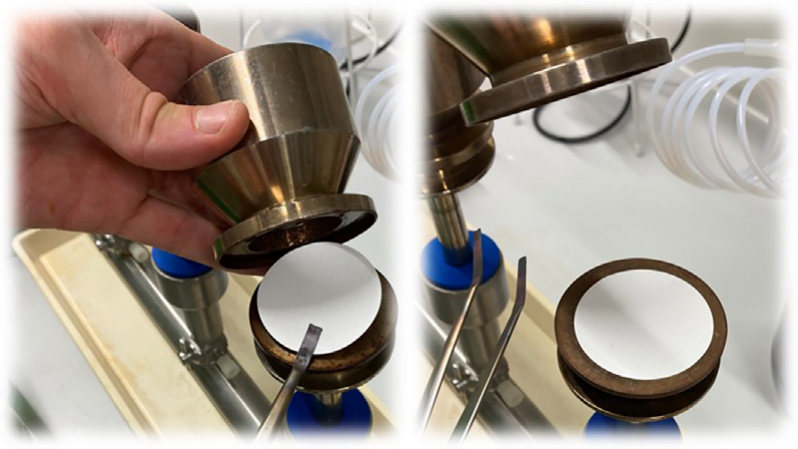
Place the filter on the sterilized filtration apparatus for each sample
11. Place the filter on an Endo Agar plate for cultivating the pathogens. Troubleshooting 4.
  a. Incubate the plates for 24 h at 37°C.
  b. Afterwards count the colonies (e.g., Figure 5) and calculate the CFU/mL for both strains.

**Figure 5:**
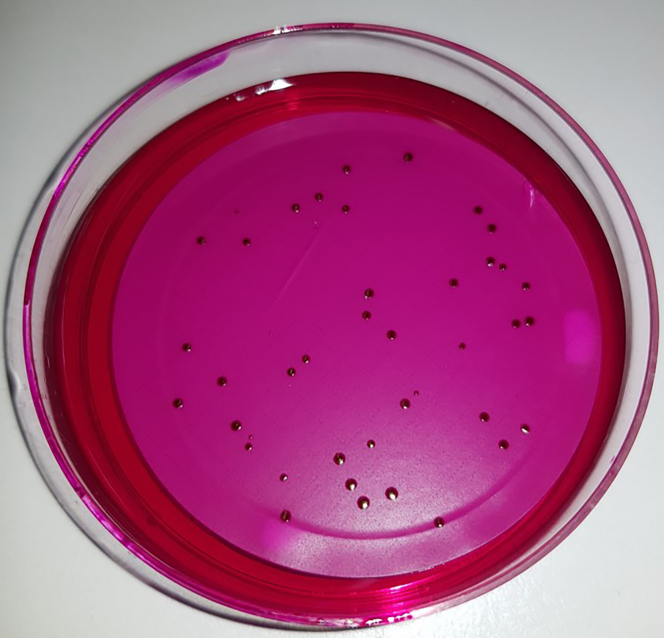
Counting CFU/mL After Filtration and incubation of the agar plates count all the colonies on the plates to calculate the CFU/mL.

***Optional:*** If you work under different conditions or if you want to test new strains, vary your collection time points to gain significant results.

***Note:*** Further down the period do duplicates using 10 mL (1:10) of the samples as higher colony counts may occur.

### Step four: Validation of strains

**Timing: 1–1.5 h**

The purpose of this step is to confirm the respected colonies of the artificial contaminated water samples.^3^^,^^11^

12. Select different single colonies on the plate for the identification and transfer them on TSA agar to get pure cultures.
  a. First, confirm your respected strains checking the morphological pattern.
  b. Take an inoculation loop to test whether your single colony is oxidase positive or negative.
  c. Pick the same colonies from pure cultures and place them on the well of the MS-chip.

***Note:*** For the determination of the respected strains, MALDI TOF analyses can be performed.

## Expected outcomes

After the bacterial strains adapted to the minimal nutrient conditions of the bottled water they started growing in mineral water. At day one, we found a reduced CFU/mL in sparkling mineral water (*E. coli*). Moreover, *E. coli* showed a decreased lifespan in mineral water without CO_2_ due to the fact of low nutrition in the water. In contrast, the CFU/mL for *P. aeruginosa* in mineral water without CO_2_ escalates after an incubation of 31 days. However, the low pH values due to the carbonation of the water and the damaging effects of CO_2_ lead to decreased CFU/mL (Table 2) of both strains in sparkling water.

**Table 2:** Mean values for CFU/mL of *E. coli* and *P. aeruginosa* in mineral water with and without carbonization

Strain	*E. coli*		*P. aeruginosa*	
Time [day]	Bottled water	Sparkling bottled water	Bottled water	Sparkling bottled water
1	45 ± 1	28 ± 2	86 ± 2	94 ± 4
2	62 ± 4	4 ± 0	170 ± 22	0
7	95 ± 6	0	120 ± 3	0
14	85 ± 2	0	220 ± 6	0
21	28 ± 4	0	380 ± 7	0
31	2 ± 0	0	76000 ± 0	0

## Limitations

Our method of artificially contaminated water is not investigating the interaction of different pathogens in a consortium. Therefore, the CFU of the pathogenic strains or any other bacterial strains in the contaminant solution has to be adjusted. Our protocol determines the CFU/mL of the single pathogen with no data of interaction or biofilm formation.

## Troubleshooting

### Problem 1

If the sample is containing high amounts of chloride ions (≥3 g/L) or a carbonate hardness ≥30°dH (German Hardness degree), see the solution below.

### Potential solution

Prepare a dilution, e.g., 1:2 or 1:5, (step 3) to gain valid values for TOC.

### Problem 2

Cross-sensitivities or insufficient resolutions are rarely observed, but can occur at large concentration differences between the anions in step 5.

### Potential solution

Proceed with the instruction of EN ISO 10304-1:2009.

### Problem 3

If the water sample touches the screw cap it can consequently lead to cross-contaminations, especially in Step 8.b.

### Potential solution

Mix the inoculated water carefully to avoid any contaminations.

### Problem 4

**Figure 6:**
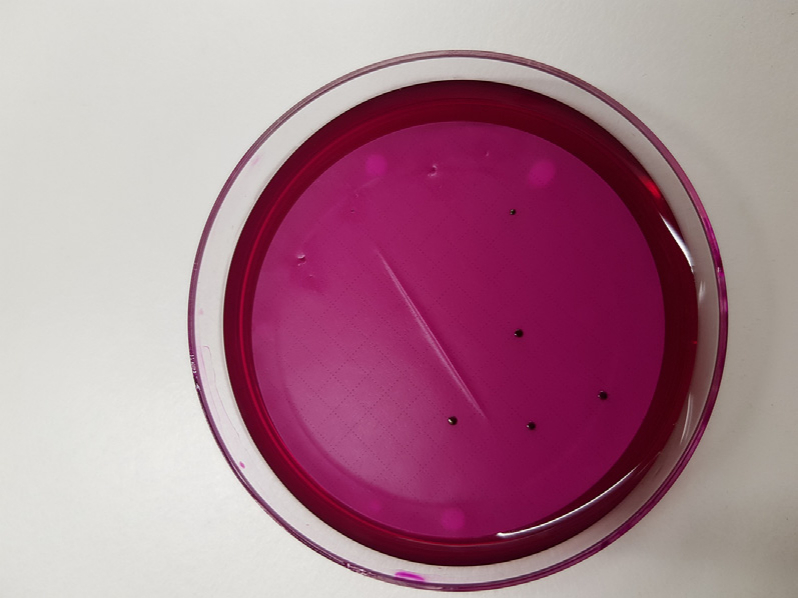
Avoid air inclusions and folding in the membrane In the case of air inclusions, you may lose CFU, since the nutrition of the agar cannot diffuse through the membrane.

Transferring the membrane to the agar plate is fussy, referring to step 11. The transfer membrane disc should have no cracks or air inclusions among. Moreover, any of those defects will lead to loss of CFU and consequently a wrong outcome (Figure 6).

### Potential solution

• It is important to take forceps with no bended tips or any damaged parts. Since the forceps are broken, you will easily damage the membrane.
• The membrane should be placed down in a constant movement and don't jerk. Otherwise, it will cause air inclusions or a rippled membrane surface (e.g., Figure 5).

## Resource availability

### Lead contact

Further information and requests for resources and reagents should be directed to and will be fulfilled by the lead contact, Dr. Michael Schalli (michael.schalli@medunigraz.at).

### Technical contact

Questions about the technical performance should be sent to the technical contact, Sabine Platzer (sabine.platzer@medunigraz.at).

### Materials availability

This protocol did not generate any new resources, all the materials are commercially available.

### Data and code availability

The raw data of this investigation and analyses are available on https://doi.org/10.1016/j.heliyon.2023.e21634. If any additional data or information is needed regarding this investigation, please contact the lead contact.
